# Modification and Compounding of CaMgAl-Layered Double Hydroxides and Their Application in the Flame Retardance of Acrylonitrile-Butadiene-Styrene Resin

**DOI:** 10.3390/polym11101623

**Published:** 2019-10-08

**Authors:** Bai-nian Wang, Ming-yang Chen, Bao-jun Yang

**Affiliations:** School of Chemistry Engineering, Hefei University of Technology, Hefei 230009, China; 284855884@163.com (B.-n.W.); cmy704168103@163.com (M.-y.C.)

**Keywords:** ABS, flame retardant, layered double hydroxides, sodium oleate, expanded graphite, ammonium

## Abstract

CaMgAl-layered double hydroxides (CaMgAl-LDHs) were synthesized by a co-precipitation method to prepare sodium oleate-modified, borate-intercalated CaMgAl-LDHs (O-CaMgAl-LDHs) using in-situ intercalation and modification, and the LDHs samples were characterized by X-ray diffraction (XRD), Fourier transform infrared spectroscopy (FT-IR), field emission scanning electron microscopy (FESEM), and thermal gravimetric analysis (TGA). The FESEM observations showed that the as-prepared CaMgAl-LDHs had a lamellar structure with a particle size of 200~500 nm, while the O-CaMgAl-LDHs had a plate-like structure with a particle size of about 100 nm. TGA showed that O-CaMgAl-LDHs resulted in higher thermal stability at high temperature compared to CaMgAl-LDHs. O-CaMgAl-LDHs/ABS composites were prepared by adding O-CaMgAl-LDHs to acrylonitrile-butadiene-styrene resin (ABS) to test the resulting flame retardancy and mechanical properties, and the results showed that the limiting oxygen index (LOI) could increase from 18% to 26%, while the mechanical properties decreased significantly when the added fraction was 40% (relative to ABS). O-CaMgAl-LDHs, ammonium polyphosphate (APP) and expandable graphite (EG) were added into the ABS to prepare ABS composites, and the effects of different compositions on the flame retardancy and mechanical properties of the ABS composites were investigated. The results showed that, when adding 5 g of O-CaMgAl-LDHs, 1 g of APP, and 14 g of EG into 40 g of ABS, the LOI of the ABS composite reached 28.8%, and the composite prepared could meet the V-0 grade requirements of the UL-94 combustion test, while the flexural strength decreased only 21.9% compared to pure ABS, the smallest decrease compared to all of the other composites.

## 1. Introduction

ABS resin is a rubber-toughened thermoplastic of acrylonitrile, butadiene, and styrene and has many excellent properties because of the diversity of its components, such as chemical resistance, simple processing, good mechanical properties, and other characteristics [1,2]. However, ABS resin is extremely inflammable (LOI is about 19), which limits its application in many aspects. Therefore, finding a suitable flame retardant to overcome this shortcoming has become a hot topic of current research [3,4,5,6,7]. At present, most flame retardants for ABS are based on a halogen and have high efficiency, but some countries have prohibited the use of halogen flame retardants due to their pollution of the environment when combusting. In this respect, the use of nanoclays has become a promising candidate [8].

Layered double hydroxides (LDHs), also known as hydrotalcites, are a kind of synthetic mineral found in association with layered silicates (cationic clays). LDHs are layered compounds consisting of brucite-like (positively charged), exchangeable inter-layer anions, and water molecules, and are also named anionic clays. The ideal chemical composition can be expressed as [M^2+x^N^3+^(OH)_2x+2_]^+^[An^−1/n^·mH_2_O], where M^2+^ represents a divalent metal cation, such as Mg^2+^, Ca^2+^, Cu^2+^, Zn^2+^, etc., N^3+^ represents a trivalent metal cation, such as Al^3+^, Fe^3+^, Cr^3+^, etc., A^n−^ represents the interlayer anion, such as CO_3_^2−^, NO_3_^−^,SO_3_^2−^, Cl^−^ etc., and *x* represents the molar ratio of the M^2+^ to N^3+^, usually ranging from 2 to 4 [9,10]. LDHs have been widely used in many fields, such as catalysis, ion exchanging, absorption, and flame retardance, because of their excellent anion exchange property and thermal stability [11,12,13]. LDHs show obvious flame retardance and smoke suppression since they lose inter-layer water and intercalated anions, transferring into complex metal oxides, when combusting. In the process, LDHs absorb a high amount of heat, dilute the oxygen concentration, and form a carbon covering on the surface of composites, thus protecting the polymer bulk from exposure in the air [14]. LDHs have been widely used in materials attributed to its halogen-free, environmental characteristics [15].

ABS produces black smoke when combusting, while borates have a significant smoke suppressant property. By adding a borate into the LDH precursor layers, the intercalated products were expected to have excellent flame retardant and smoke suppressant properties and thus to be used in the preparation of flame retardant composites as an efficient, inorganic, halogen-free flame retardant. In our experiments, borate was intercalated into the layers of the CaMgAl-LDHs precursor to suppress smoke, and the surfaces of the borate-intercalated LDHs were then modified by sodium oleate to enhance their compatibility with the ABS. Finally, the surface-modified borate-intercalated CaMgAl-LDHs, APP and EG were added to ABS to test the flame retardant and mechanical properties.

## 2. Materials and Methods

### 2.1. Materials

Calcium nitrate, magnesium nitrate, aluminum nitrate, sodium oleate, and boric acid, all with analytical purity, were obtained from the Shanghai Chemical Reagent Co. of the Chinese National Pharmaceutical Group. EG was bought from Qingdao Teng Shengda Carbon Machinery Co. Ltd. (Qingdao, China), and ABS was bought from Qi Mei Co. Ltd. (Taiwan, China).

### 2.2. Preparation of CaMgAl-LDHs

The LDHs were prepared by co-precipitation [16,17,18]. The experimental procedures were as follows: 0.06 mol Ca(NO_3_)_2_·4H_2_O, 0.12 mol Mg(NO_3_)_2_·6H_2_O, and 0.06 mol Al(NO_3_)_3_·9H_2_O were added into 100 mL of deionized water to prepare a salt solution, and 0.48 mol NaOH was added into 100 mL of deionized water to prepare an alkali solution (B). Keeping the reaction temperature at 25 °C, the alkali solution (B) was dropped into the salt solution (A) 1 drop per second until the pH reached 10. Stirring was continued for 2 h while dynamic crystallization occurred, followed by static crystallization for 20 h with no stirring. After that, the reaction mixture was filtered, and the filter cake was washed with deionized water and then dried for 6 h at 100 °C to obtain the CaMgAl-LDHs samples.

### 2.3. Preparation of Borate Intercalated CaMgAl-LDHs

The washed, undried LDH filter cake was dispersed into a saturated boric acid solution at 95 °C with vigorous stirring for 6 h and then aged for 20 h with no stirring. After that, the reaction mixture was filtered, and the filter cake was washed with deionized water and dried for 6 h at 100 °C, yielding the borate-intercalated CaMgAl-LDHs samples (B-CaMgAl-LDHs). 

### 2.4. Preparation of CaMgAl-LDHs with Sodium Oleate Modified Borate Intercalation

The washed, undried filter cake of B-CaMgAl-LDHs was added into a 2% sodium oleate solution and stirred for 1.5 h at 65 °C. After that, the reaction mixture was filtered, and the filter cake was washed with deionized water and dried for 6 h at 100 °C to obtain the sodium oleate-modified, borate-intercalated CaMgAl-LDHs samples (O-CaMgAl-LDHs).

### 2.5. Preparation of Composites

O-CaMgAl-LDHs and ABS were mixed to prepare the O-CaMgAl-LDHs/ABS composites. The O-CaMgAl-LDHs, APP, EG, and ABS were mixed in different ratios (shown in Table 1) to prepare the O-CaMgAl-LDHs/APP/EG/ABS composites. The preparation process was as follows: the mixture was melt blended by a twin-roll mill (JTC-100, Chengde Testing Machine Co., Ltd., Chengde, China) at 180 °C for 10 min, and subsequently compression molded at 195 °C under a pressure of 10 Kpa as 4 mm thick films, followed by cold pressing at room temperature on a flat vulcanizer (XLB-D400, Shanghai Rubber Mechanics Factory, Shanghai, China), to obtain the ABS composites. They were held at room temperature for 24 h and then cut into standard sample bars (length l = 100 ± 2 mm, width b = 10.0 ± 0.2 mm, and thickness h = 4.0 ± 0.20 mm) for testing.

### 2.6. Testing and Characterization

XRD characterization of the LDHs was carried out on a D/Max2500 X-ray diffractometer (Rigaku Co., Tokyo, Japan, Cu*K*_α_, λ = 0.1540 nm). The infrared spectra of the samples were measured with a Nicolet 6700 Fourier transform infrared spectrometer (FTIR, Thermo Nicolet Corp., Madison, Wisconsin, USA, range of wavenumbers: 400~4000 cm^−1^, resolution: 0.09 cm^−1^, KBr), and their microscopic morphology was observed with an SU8020 scanning electron microscope (FESEM, acceleration voltage 5 KV, Hitachi Co., Tokyo, Japan). The thermal stabilities of the samples prepared were characterized by a TG209-F3 type thermogravimetric analyzer (German Relaxation Instrument Manufacturing Co. Ltd., Selb, German) (N_2_ atmosphere, at a heating rate of 10 °C/min, test temperature 35~700 °C). The limiting oxygen index of the composites was detected by an HC-2 oxygen index instrument (Nanjing Jiangning District Analysis Instrument Factory, Nanjing, China) according to GB/T2406.2-2009. The UL-94 vertical burning tests were carried out on a CZF-3 vertical combustion tester (Nanjing Jiangning District Analysis Instrument Factory, Nanjing, China). The flexural strength, tensile strength, and elongation at break were measured on a CMT4304 electronic universal testing machine (Shenzhen Xinsi Si Material Inspection Co. Ltd., Shenzhen, China) according to GB/T9341-2008 and GB/T1040-92, respectively.

## 3. Results

### 3.1. Structure and Morphology

**Figure 1:**
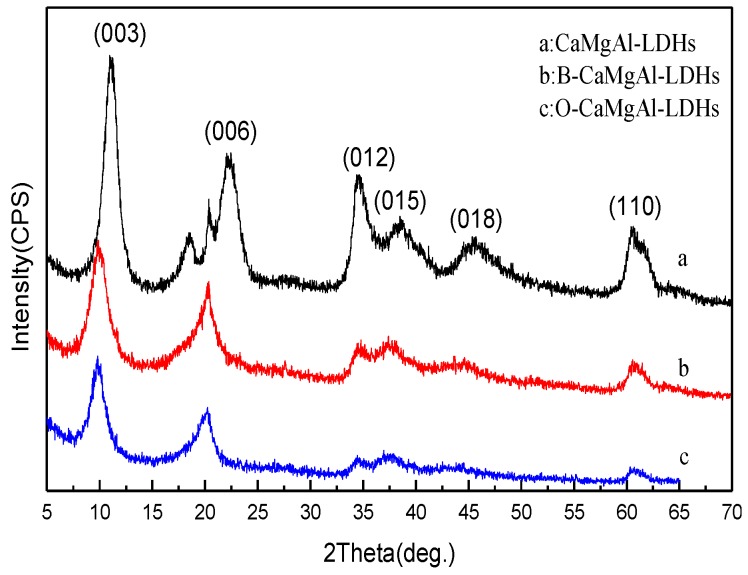
XRD patterns of CaMgAl-LDHs, B-CaMgAl-LDHs, and O-CaMgAl-LDHs.

Figure 1 shows the XRD patterns of CaMgAl-LDHs, B-CaMgAl-LDHs, and O-CaMgAl-LDHs. In the patterns, all of the synthesized LDHs exhibited strong diffraction peaks at angles that can be indexed as the (003), (006), and (012) planes of the LDH crystals. In addition, at a high 2θ angle, all the samples exhibited a diffraction peak indexed as the (110) plane, which indicated the mono-crystalline structure of the LDHs [17]. In addition, in Figure 1b and c, all the diffraction peaks decreased in intensity gradually and broadened, indicating the decreased grain size of the LDHs with the modification by boric acid and sodium oleate [19,20,21]. It can also be seen that the characteristic diffraction peaks (003) and (006) of the layered structures of B-CaMgAl-LDHs and O-CaMgAl-LDHs moved to lower 2θ angles and decreased in intensity compared to those of the CaMgAl-LDHs precursor, but remained equidistant between peaks (003) and (006), demonstrating that anions with larger volumes entered the interlayer [22], which indicated that the borate groups successfully replaced the nitrate group.

**Figure 2:**
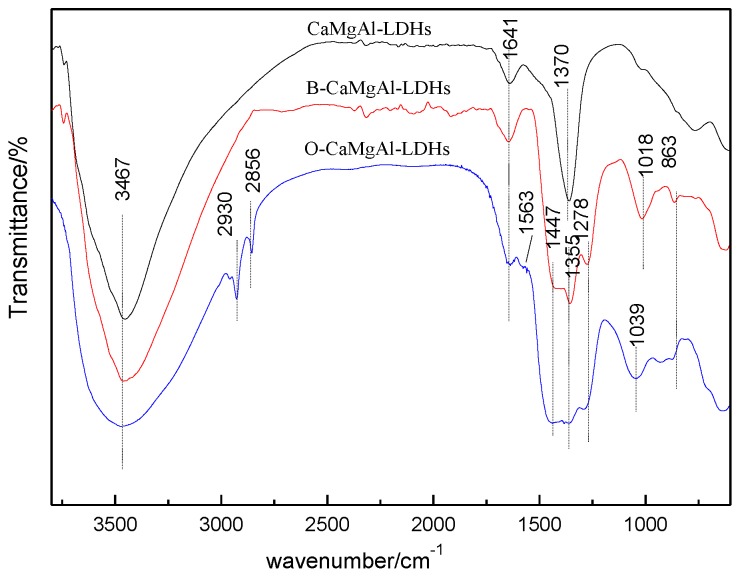
FT-IR patterns of CaMgAl-LDHs, B-CaMgAl-LDHs, and O-CaMgAl-LDHs.

Figure 2 shows the FTIR spectra of CaMgAl-LDHs, B-CaMgAl-LDHs, and O-CaMgAl-LDHs. All the samples exhibited absorption peaks at 3467 and 1641 cm^−1^ corresponding to the stretching vibrations of the hydroxyl groups ν-OH on the LDHs layers and the bending vibrations of the interlayer δ-OH. The absorption peaks at 1370 cm^−1^ in curve a and at 1355 cm^−1^ in curves b and c correspond to the stretching vibrations (ν_3_) of NO_3_^−^ [23]. The absorption peaks at 1447 and 1355 cm^−1^ can be attributed to the anti-symmetric stretching vibrations of BO_3_^3^^−^ [23]. The absorption peaks at 1018 and 1039 cm^−1^ in curves b and c can be attributed to the anti-symmetric stretching vibrations of BO_4_^5−^ [23]. The absorption peak at 863 cm^−1^ can be attributed to the surface bending vibrations of B-OH [24]. These latter five peaks showed that borate was inserted into the CaMgAl-LDHs layers, agreeing with the XRD results analysis. In addition, the infrared spectrum of O-CaMgAl-LDHs exhibited absorption peaks at 2930 and 2856 cm^−1^, corresponding to the symmetric and asymmetric stretching vibrations of C–H in a saturated alkane, respectively [25], and an absorption peak near 1563 cm^−1^, which can be attributed to the asymmetric stretching vibration peak of –COO– [26], indicating the successful modification by sodium oleate.

**Figure 3:**
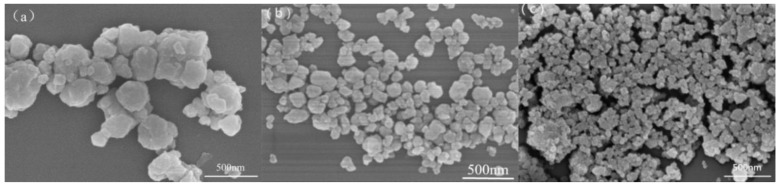
FESEM images of CaMgAl-LDHs (**a**), B-CaMgAl-LDHs (**b**), and O-CaMgAl-LDHs (**c**).

Figure 3 shows the FESEM images of CaMgAl-LDHs, B-CaMgAl-LDHs, and O-CaMgAl-LDHs. The CaMgAl-LDHs consisted of solid particles with a size of ca. 200~400 nm but seriously aggregated. The B-CaMgAl-LDHs had a similar solid structure, but with a smaller size, of about 100 nm, and little evidence of aggregation. The O-CaMgAl-LDHs appeared to consist of aggregates, of various overall size, of much smaller particles than the B-CaMgAl-LDHs.

**Figure 4:**
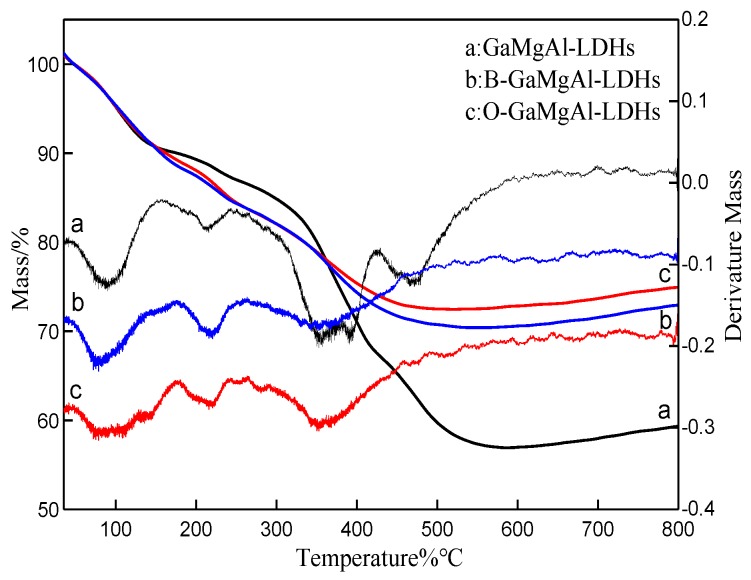
TGA/DTG curves of CaMgAl-LDHs, B-CaMgAl-LDHs and O-CaMgAl-LDHs.

Figure 4 shows the thermogravimetric curves and derivative thermogravimetric curves of CaMgAl-LDHs, B-CaMgAl-LDHs, and O-CaMgAl-LDHs. The degradation of CaMgAl-LDHs is generally divided into three stages [22]: The weight loss of the LDHs at 40~150 °C is mainly due to the removal of adsorbed water and interlayer bound water, with the plates not being destroyed in this stage. The weight loss of the CaMgAl-LDHs at 150~320 °C corresponds to the dehydroxylation reaction of the hydroxyl radicals binding with the calcium, magnesium, and aluminum on the surfaces of the layers; the laminates break up in this stage. Dehydration continued at 320~600 °C due to the decomposition of a small amount of nitrate ions between the layers, with calcium, magnesium, and aluminum oxide produced in this stage. The weight loss values of the CaMgAl-LDHs in the three stages were 9.3, 7.1, and 26.6%, respectively. As for B-CaMgAl-LDHs and O-CaMgAl-LDHs, there were only two stages, which were 40~450 °C and 450~600 °C, respectively. The weight loss values of the B-CaMgAl-LDHs and O-CaMgAl-LDHs in the two stages were 9.3 and 13.3% and 9.5 and 13.6%, respectively. The residue weight at 600 °C were 70.6% for B-CaMgAl-LDHs and 72.8% for O-CaMgAl-LDHs, which are both significantly higher than that of CaMgAl-LDHs. This is because the CaMgAl-LDHs are actually CaMgAl-NO_3_-LDHs, but the O-CaMgAl-LDHs and B-CaMgAl-LDHs are O-CaMgAl-BO_3_-LDHs and B-CaMgAl-BO_3_-LDHs. Therefore, when heated to 600 °C, the CaMgAl-NO_3_-LDHs (a) release oxynitride while the B-CaMgAl-BO_3_-LDHs (b) and O-CaMgAl-BO_3_-LDHs (c) decompose into B_2_O_3_, which remains in the residue, causing significantly higher residue weight.

### 3.2. Flame-Retardation Properties

Table 2 shows the flame retardancy and mechanical properties of the composites with the addition of various amounts of the O-CaMgAl-LDHs only. It can be seen that the LOI of the composites increased slightly with the addition of increasing amounts of O-CaMgAl-LDHs. When the addition of the LDHs was 40%, the composites had the highest LOI and could meet the requirements of the V-1 grade in the UL-94 combustion test, but the flexural strength, tensile strength, and breaking elongation were only 26.5, 30, and 20.9% relative to those of pure ABS. It is thought that the physical mixing causes poor connection between the flame retardant particles and ABS. Moreover, inorganic LDHs have poor compatibility with organic ABS. These two reasons may cause the decline in the mechanical properties of the ABS composites. In a word, when O-CaMgAl-LDHs were added alone to ABS, as a flame retardant, the effect on the mechanical properties was not desirable and the improvement of the LOI was only slight.

Thus, the addition of only O-CaMgAl-LDHs to ABS when using it as a flame retardant would be inappropriate, since it resulted in a rapid decrease in mechanical properties of the composites. Therefore, on the basis of these experiments, we carried out further studies on the combination of LDHs and other flame retardants, ammonium polyphosphate (APP) and expandable graphite (EG), to improve both the flame retardance and mechanical properties of the ABS composites.

**Figure 5:**
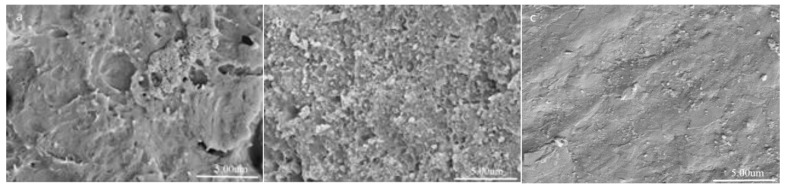
FESEM images of the fracture surfaces of ABS_1_, ABS_2_, and ABS_4_.

Figure 5 shows the FESEM images of the tensile fracture surfaces of O-CaMgAl-LDHs/ABS (ABS_1_), O-CaMgAl-LDHs/APP/ABS (ABS_2_), and O-CaMgAl-LDHs/APP/EG/ABS (ABS_4_). The fracture surface of the O-CaMgAl-LDHs in ABS_1_ (Figure 5a) was not very uniform, looking like a discontinuous broken membrane with a few small bright particles appearing on it. Based on our experience, we suggest that these particles might be LDHs and/or APP not mixed with ABS. When adding APP and LDHs (Figure 5b), the tensile fracture surface was relatively flat, but with many small particles indicating better dispersion of the flame retardants but with no adhesion to the matrix. When adding O-CaMgAl-LDHs, APP, and EG together (Figure 5c), the fracture surface was very smooth with a few small particles on the surfaces, indicating an even better dispersion of the flame retardants.

Table 3 shows the flame retardancy (FR, LOI, and UL-94) and mechanical properties of the ABS and ABS composites. It can be seen that the LOI of the composites with added EG increased significantly compared to the other ABS composites, with the same total additions of 20 g. The LOI and flexural strength of ABS_4_ were significantly higher than those of the other ABS composites for the same total weight of additions, while the tensile strength was higher than for ABS_1_ but lower than for ABS_2_. With the increase of the amount of EG, the flexural strength of ABS_4_ was higher than that of ABS_3_; the LOI of the composites increased continually with the increase of the relative amount of EG, reaching a maximum of 28.8%. When adding 14 g of EG, the as-prepared composite ABS_4_ met the requirements of the V-0 grade in the UL-94 combustion test, while all of the composites with added EG and APP in addition to the LDHs showed better mechanical properties compared to those with added LDHs only.

As for the action mechanism of the flame retardant, it is thought that the flame retardant can interfere physically or chemically with the various processes involved in polymer combustion [27]. In this experiment, LDHs will absorb heat and form porous metal hydroxide when combustion happens. Inert gases such as H_2_O, NH_3_, CO_2_, and N_2_ released by LDHs and APP can dilute the concentration of O_2_ around the composite delaying the combustion [28,29].

When adding EG, it will expand during the combustion, forming a “vermicular” protective carbon layer covering the surface of the composite, which enhances the ability to isolate oxygen and to cut off heat transfer, thus improving the flame retardant performance of the ABS composites [30].

Therefore, we deem that LDHs, APP, and EG have synergistic interactions, remarkably improving the flame retardant performance of the ABS composite.

**Figure 6:**
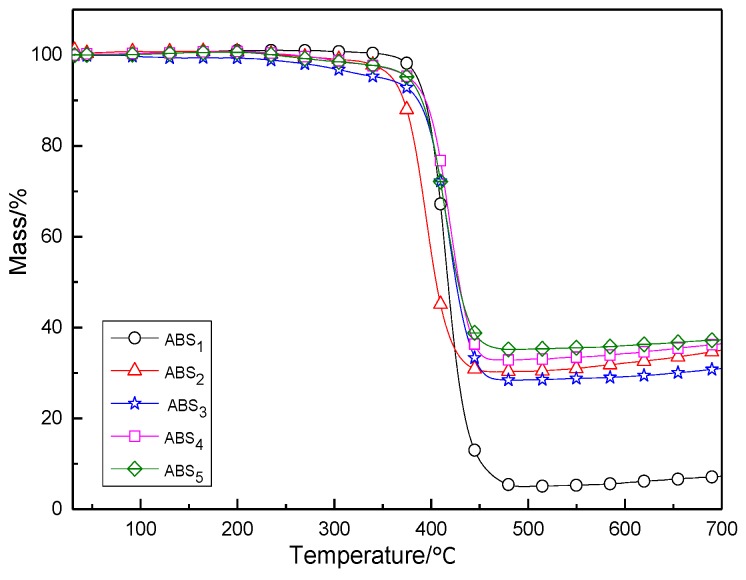
TGA curves of ABS and ABS composites.

Figure 6 shows the weight loss curves of the five samples. It can be seen that both pure ABS resin and the composites began to degrade at about 380 °C and were degraded completely at 500 °C. The weight loss of ABS was 95%, with only a very small amount of carbon residue, indicating that the majority of the resin decomposed as volatile gases of small molecules or H_2_O. The weight losses of the composites were 69.7, 71.5, 67.0, and 64.7%, respectively, for ABS_1_, ABS_2_, ABS_3_, and ABS_4_. The composites with added EG had the lowest weight losses with much higher residues than ABS, because it produced a dense carbon layer adhering to the surface of the ABS composite when combusting and retarded the decomposition of the ABS, causing the increase of the carbon residue.

**Figure 7:**
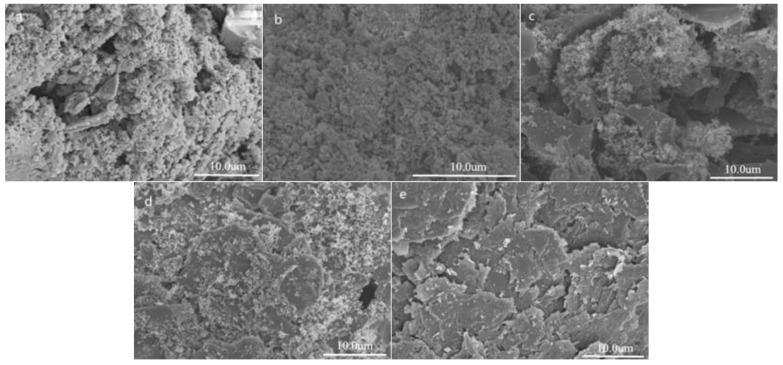
FESEM images of the carbon residues of ABS (**a**), ABS_1_ (**b**), ABS_2_ (**c**), ABS_3_ (**d**), and ABS_4_ (**e**).

Figure 7 shows the FESEM images the surface of ABS, and the ABS_1_, ABS_2_, ABS_3_, and ABS_4_ composites after combustion. It can be seen that the surface of ABS after combustion was loose and porous. When adding O-CaMgAl-LDHs alone, the surface of the ABS_1_ after combustion showed a more compact carbon residue and metal oxides. When adding O-CaMgAl-LDHs and APP, the carbon residue on the surface of ABS_2_ during combustion formed a carbon layer. When adding CaMgAl-LDHs, APP, and EG, the ABS_4_ carbon layer became denser, which could protect the polymer matrix from the erosion of the flame, delaying the heat transfer between the gas and condensed phase and thus achieving the flame retardance effect. The denser the carbon layer that was formed, the more effective the protection of the polymer matrix became, thus having a better flame retardance effect. From the above analysis, we can see that LDHs, APP, and EG had a good, synergistic flame retardant effect on the ABS composites, but, even for the optimum amounts, had a detrimental effect on the mechanical properties.

## 4. Conclusions

CaMgAl-LDHs were synthesized via a co-precipitation method, and O-CaMgAl-LDHs with diameters of about 100 nm were prepared by borate intercalation and sodium oleate wet surface modification.

The O-CaMgAl-LDHs were added to ABS to prepare ABS composites. The flame retardancy tests showed that the LOI increased with the increase of the O-CaMgAl-LDHs; when the addition of O-CaMgAl-LDHs was 40%, the limiting oxygen index (LOI) reached 26%, but the sample’s elongation at break, tensile strength, and other mechanical properties decreased significantly.

O-CaMgAl-LDHs/ABS, O-CaMgAl-LDHs/APP/ABS, and O-CaMgAl-LDHs/APP/EG/ABS composites were prepared using O-CaMgAl-LDHs, APP, and EG as flame retardants to investigate their synergistic flame retardant effects and their effect on the mechanical properties. The results showed that the LOI of the ABS_4_ composites could reach 28.8%, with this composite meeting the requirements of the V-0 grade in the UL-94 combustion test, indicating that LDHs, APP, and EG had a good synergistic flame retardant effect, while the mechanical properties of the composite decreased the least compared to all the other composites.

## Figures and Tables

**Table 1:** Formulations of the flame retardant ABS with added O-CaMgAl-LDHs, APP, and EG

Sample	ABS(g)	LDHs(g)	APP(g)	EG(g)
ABS	60	0	0	0
ABS_1_	40	20	0	0
ABS_2_	40	15	5	0
ABS_3_	40	10	3	7
ABS_4_	40	5	1	14

**Table 2:** The flame retardant and mechanical performances of the ABS composites with various amounts of added O-CaMgAl-LDHs.

Mass Fraction of O-CaMgAl-LDHs/%	LOI/%	UL-94	Flexural Strength/MPa	Tensile Strength/MPa	Breaking Elongation/%
0	19	No	85.21	46.33	21.3
10	22.5	No	77.17	28.57	9.12
20	23.5	No	65.31	19.1	8.48
30	25	No	49.12	15.5	6.42
40	26	V-1	22.56	13.9	4.45

**Table 3:** Flame retardance and mechanical properties of the ABS composites with added O-CaMgAl-LDHs, APP, and EG.

Sample	LOI/%	Flexural Strength/MPa	Tensile Strength/MPa	Breaking Elongation/%	UL-94
ABS	19.0	85.21	46.33	21.30	No
ABS_1_	25.2	44.52	14.4	5.74	No
ABS_2_	25.8	52.21	24.85	9.34	No
ABS_3_	28.0	57.26	18.00	8.46	V-1
ABS_4_	28.8	66.59	17.35	12.99	V-0
